# Health inequalities: an analysis of hospitalizations with respect to migrant status, gender and geographical area

**DOI:** 10.1186/s12914-014-0032-9

**Published:** 2015-02-07

**Authors:** Chiara de Waure, Stefania Bruno, Giuseppe Furia, Luca Di Sciullo, Serena Carovillano, Maria Lucia Specchia, Salvatore Geraci, Walter Ricciardi

**Affiliations:** Department of Public Health, Università Cattolica del Sacro Cuore, L.go Francesco Vito 1, 00168 Rome, Italy; Consiglio Nazionale dell’Economia e del Lavoro, Rome, Italy; Area sanitaria Caritas di Roma, Rome, Italy

**Keywords:** Inequalities, Immigrant, Hospitalization, Gender, Citizenship, Italy

## Abstract

**Background:**

The quality of care includes several aspects which may be influenced by social-economic status.

This study analyzes hospitalizations for several conditions, such as chronic diseases, cancer and appendectomy, in Italians and immigrant people living in Italy with the aim to evaluate possible inequalities in the quality of health care services due to migrant status, gender and geographical macro-areas (Northern, Central, Southern Italy).

**Methods:**

The data source of hospital discharges for stroke, myocardial infarction, chronic liver disease, cervical cancer, mastectomy and appendectomy was the Ministry of Health. ICD 9 codes were used for data collection. Crude and standardized hospitalization rates per 100.000 were calculated. Italian resident population and an estimate of immigrants living in Italy were used as denominators while standardization was done with respect to the European population. The data we used covers the 2006–2008 period.

**Results:**

Immigrants showed significantly higher hospitalization rates for stroke, cervical cancer and appendectomy and significantly lower hospitalization rates for chronic liver diseases and mastectomy. Males showed significantly higher hospitalization rates than females for myocardial infarction, chronic liver diseases and appendectomy. Notwithstanding, differences related to migrant status and gender varied according to geographical macro-area. With respect to that, Southern Italy showed significantly higher hospitalization rates for stroke, myocardial infarction and chronic liver diseases and significantly lower hospitalization rates for mastectomy and appendectomy.

**Conclusions:**

The results of this study may reflect inequalities in the quality of health care, in particular in primary and secondary prevention, access to specialized care and inappropriateness, due to migrant status and gender. Also, differences between macro-areas suggest heterogeneities in the integration policies and the promotion of immigrants’ health. Research should be endorsed in this field in order to further describe inequalities and their reasons and in the light of supporting policies development.

**Electronic supplementary material:**

The online version of this article (doi:10.1186/s12914-014-0032-9) contains supplementary material, which is available to authorized users.

## Background

Health inequalities are systematic differences in health status between groups of people belonging to different socioeconomic positions within countries and between countries. They may be considered inequities if they are unfair and avoidable. The absence of equity determines the deterioration of health conditions, especially in socially disadvantaged groups, resulting in a social gradient of health [1,2].

Social determinants of health play an important role in determining inequalities both in the developmental phase of life [3,4] and in adulthood. Regarding the latter, limitations/lack of access to social goods, which may be associated to gender and race/ethnicity, may have an impact on health. In particular, in recent years, inequalities due to migrant status have been paid a growing interest and attention both in the literature and in declarations released by international political-institutional organizations [5]. In September 2007, the Conference “Health and Migration in the EU: better health for all in an inclusive society” established the importance of migrants’ universal access to care as an essential element for social, economic and political development of countries as well as for the promotion of human rights. Indeed, the promotion of integration and of an equal access to care is an urgent instance in Public Health [6]. The same “Bratislava Declaration on health, human rights and migration” [7] established that health interventions for migrants, including Public Health ones, should be implemented in order to promote the well-being of all people and the integration of migrants. In accordance to this, in March 2011, the European Parliament approved a resolution on reducing health inequalities in the European Union [8], and the Member States were invited to address inequalities in access to health care for undocumented immigrants, pregnant women and children. It was emphasized that “Health inequalities are not only the result of a multitude of economic, environmental or lifestyle factors, but they also depend on access to health care services”. Nevertheless the Member States have not yet achieved a common direction about integration policies for migrants, although the right to health is profoundly rooted, as enshrined in the Declaration of Alma Ata which incorporates a social dimension in governmental policies, with particular attention to disadvantaged groups such as women, elderly people, irregular immigrants, refugees and asylum seekers [9].

In Italy, over the last 30 years, the presence of non-Italian citizens has increased. In 2011 around 4,000,000 individuals were regularly present in Italy, which represented 8,6% of the national population, compared to a European average of 6,6%. An heterogeneity of immigrant distribution in the country may be appreciated with more than half of immigrants living in North, followed by Central and Southern Italy [10,11]. Immigrants are a young population, represented about half by women, with a significant presence of minors (about one million). They come from over 190 different countries. In particular, 27,4% come from countries that are part of the European Union, 23,4% from non-Community European Countries, 22,1% from Africa, 18,8% from Asia, 8,3% from America and the remaining from Oceania [12,13].

Since the 1990s, Italian national health policies and laws allow regular non-Italian citizens the access to the National Health Service (NHS). Furthermore, the delivery of care is provided also to those who are temporarily irregular. Nevertheless the heterogeneity of health policies adopted by individual regions determines inequalities in the availability of services [14,15] and phenomena, such as high rates of voluntary interruption of pregnancy [10], which deserve more attention to integration policies. With this respect, the knowledge of the current access to health care and of the quality of health services is fundamental in order to support the decision-making process.

This study analyzes hospitalizations for several conditions, such as chronic diseases, cancer and appendectomy, in Italians and immigrant people living in Italy. The final aim is to allow evaluating potential differences in the quality of health care services, which is made up of several aspects, among which access to healthcare [16], due to migrant status as well as to gender and geographical area. The new aspects of this cross-sectional study are the assessment of health inequalities through the analysis of hospitalization data, as a proxy of the quality of health care services, and the attempt to release an estimate of immigrants’ hospitalization rate which takes into account irregular people also.

## Methods

### Study design and population

In order to assess health inequalities due to migrant status, gender and geographical area, a repeated cross-sectional study with secondary data-analysis was carried out in the time period 2006–2008 in Italy. Hospitalizations (ordinary admissions) were selected as a proxy of the quality of health care services with reference to both primary and secondary care. In particular, in order to assess inequalities in the management of diseases subjected to preventive interventions as well as inappropriateness, the following index conditions were chosen on the basis of both international and national literature and previous experience:

- stroke, myocardial infarction, cirrhosis and chronic liver diseases as a proxy of the impact of primary preventive interventions [17-20],
- cervical cancer and mastectomy as an indicator of the impact of both primary and secondary preventive initiatives [21],
- appendectomy for the assessment of the risk for surgical inappropriateness [21,22].

Data on hospitalizations were requested to the Italian Ministry of Health using The International Classification of Diseases, Ninth Revision, Clinical Modification (ICD-9-CM) codes (Table 1), stratified by gender, migrant status, age class (0–14, 15–24, 25–49, 50–64, 65+), region of discharge and year. With respect to migrant status, citizenship was used to distinguish between Italian and immigrant population.

**Table 1:** **Indicators grouped by dimensions and ICD 9 CM codes**

**Dimension**	**Indicators**	**ICD 9 CM**
**Chronic diseases**	Stroke	434.01, 434.11, 434.91, 431
	Myocardial infarction	410.xx
	Cirrhosis and chronic liver diseases	070.22, 070.23, 070.32, 070.33
		070.44, 070.54, 571.0, 571.2, 571.40, 571.41, 571.49
**Cancers**	Cervical cancer	180.xx, 233.1
	Mastectomy	85.23, 85.4, 85.22
**Inappropriateness**	Appendectomy	47.0

The population at risk for each year was obtained from the National Institute for Statistics (ISTAT) official website and through a stepwise calculation with respect to Italian and immigrant population, respectively. With regard to this latter, the estimate allowed considering people living in Italy both regularly and irregularly. The estimate took into account:

- ISTAT data on regular immigrant population,
- valid permits of residence delivered by the Ministry of Internal Affairs each year,
- foreign minors born in Italy each year,
- accepted requests for legal residence permit each year,
- a 10% of underestimation of irregular people living in Italy provided by the Foundation for Initiatives and Studies on Multi-Ethnicity.

### Statistical elaboration of data

Crude and standardized (for European population) hospitalization rates per 100.000 were calculated stratified by migrant status and gender. Furthermore, stratification by geographical macro-area was provided with respect to the following criteria:

- Northern Italy: Emilia-Romagna, Friuli-Venezia Giulia, Liguria, Lombardia, Piemonte, Trentino-Alto Adige, Valle d’Aosta and Veneto,
- Central Italy: Lazio, Marche, Toscana and Umbria,
- Southern Italy: Abruzzo, Basilicata, Calabria, Campania, Molise, Puglia, Sicilia and Sardegna.

Standardized rates and their 95% Confidence Intervals (95% CI) were considered to compare immigrants and Italians, as well as women and men, in order to avoid the bias due to the different distribution by age. 95% CIs were obtained as standardized rates ±1,96* Standard Error (SE) and SE was calculated with the following Armitage and Berry formula [23].

$$ \sqrt{\frac{{\displaystyle \sum \frac{\left({T}_ix{N_i}^2xK\right)}{n_i}}}{{\left(\sum {N}_i\right)}^2}} $$

T_i_ = crude rate for each age class

N_i_ = size of the reference population in each age class

n_i_ = size of the study population in each age class

K = multiplication factor (100.000)

The analysis of data was done descriptively with respect to the following dimensions: time differences due to migrant status, gender and geographical area.

## Results

Total hospitalizations rates significantly decreased from 2006 to 2008 and Southern Italy showed significant higher rates for both citizenships and genders. Females had always significantly higher rates than males, especially in the immigrant group, and Italians had always significant higher rates than immigrants, except for Southern Italy (Table 2). The absolute number of hospitalizations is reported in Additional file 1.

**Table 2:** **Total discharges - standardized hospitalization rates for European population (per 100.000), with 95% CI, stratified for macro-area and year**

	**Northern Italy**				**Central Italy**				**Southern Italy**			
	**Males**		**Females**		**Males**		**Females**		**Males**		**Females**	
	**Italians**	**Immigrants**	**Italians**	**Immigrants**	**Italians**	**Immigrants**	**Italians**	**Immigrants**	**Italians**	**Immigrants**	**Italians**	**Immigrants**
**2006**	14428,7 (14377,5-14480,0)	9595,4 (9287,1-9903,6)	15466,3 (15414,3-15518,3)	14058,2 (13744,6-14371,8)	14922,8 (14615,7-15229,9)	9110,4 (7532,4-10688,4)	15703,8 (15393,9-16013,6)	13590,0 (11905,9-15274,2)	25373,1 (25326,1-25420,1)	27934,5 (27626,9-28242,0)	27076,2 (27029,0- 27123,4)	41722,9 (41363,5-42082,3)
**2007**	13676,1 (13626,2-13726,0)	9100,1 (8814,4-9385,8)	14503,8 (14453,3-14554,3)	13028,5 (12744,7-13312,3)	14550,4 (14248,6-14852,3)	8911,9 (7382,5-10441,2)	15351,1 (15045,2-15657,0)	13155,8 (11535,7-14775,9)	23986,6 (23941,0-24032,1)	24448,0 (24167,5-24728,5)	25396,6 (25350,9-25442,4)	35666,9 (35339,8-35994,0)
**2008**	13378,6 (13329,4-13427,9)	9194,5 (8925,3-9463,7)	14198,7 (14148,6-14248,7)	13310,0 (13038,9-13581,2)	14298,9 (14000,9-14597,0)	9254,4 (7708,6-10800,1)	15178,5 (14874,6-15482,4)	13624,3 (12062,0-15186,5)	23073,0 (23028,5-23117,6)	24876,5 (24593,7-25159,3)	24527,0 (24482,1-24571,9)	34748,8 (34429,9-35067,7)

Tables 3, 4 and 5 report standardized hospitalization rates for chronic diseases (stroke, myocardial infarction, cirrhosis and chronic liver diseases), cancers (cervical cancer and mastectomy) and appendectomy. From 2006 to 2008 hospitalization rates for stroke, as well as for myocardial infarction, showed a decrease even though it was not significant looking at 95% CIs. Hospitalizations for chronic liver diseases significantly decreased for Italians only (Table 3). Hospitalizations for mastectomy showed a general significant decrease in both Italians and immigrants (Table 4) while, as for cervical cancer, a not significant decrease of hospitalization rates in Northern Italy was observed both in Italians and immigrants and a slight, but not significant, increase was highlighted in Central Italy, for Italians, and in Southern Italy, for both Italians and immigrants.

**Table 3:** **Chronic diseases - standardized hospitalization rates for European population (per 100.000), with 95% CI, stratified for macro-area and year**

		**Northern Italy**				**Central Italy**				**Southern Italy**			
		**Males**		**Females**		**Males**		**Females**		**Males**		**Females**	
		**Italians**	**Immigrants**	**Italians**	**Immigrants**	**Italians**	**Immigrants**	**Italians**	**Immigrants**	**Italians**	**Immigrants**	**Italians**	**Immigrants**
**Stroke**	**2006**	151,2 (146,4-156,1)	168,8 (116,8-220,8)	151,2 (146,5-155,8)	168,5 (115,9-221,1)	193,7 (160,3-227,1)	128,7 (0–370,0)	190,0 (158,2-221,8)	101,7 (0–327,6)	221,6 (217,4-225,8)	311,3 (267,3-355,2)	220,0 (216,0-224,0)	331,0 (284,8-377,3)
	**2007**	148,3 (143,5-153,0)	158,5 (110,7-206,3)	148,9 (144,4-153,5)	153,8 (106,4-201,2)	180,0 (148,1-211,8)	126,6 (0–364,6)	179,4 (148,7-210,1)	100,9 (0–321,0)	219,8 (215,7-224,0)	333,9 (289,5-378,3)	215,1 (211,2-219,0)	346,3 (300,4-392,1)
	**2008**	144,7 (140,0-149,4)	162,6 (116,7-208,5)	146,0 (141,5-150,5)	145,9 (102,9-188,9)	175,7 (144,5-206,9)	141,4 (0–387,5)	177,4 (147,0-207,9)	107,1 (0–320,1)	210,1 (206,0-214,1)	307,6 (266,6-348,6)	208,0 (204,1-211,8)	321,9 (278,1-365,8)
**Myocardial infarction**	**2006**	250,5 (244,2-256,9)	266,5 (202,8-330,1)	129,8 (125,5-134,1)	119,5 (75,9-163,1)	276,7 (236,5-317,0)	231,0 (0–549,7)	130,7 (104,2-157,2)	127,7 (0–396,5)	265,5 (260,9-270,2)	235,8 (198,5-273,2)	117,8 (114,9-120,8)	140,5 (109,3-171,7)
	**2007**	243,8 (237,6-250,0)	244,6 (187,6-301,5)	129,6 (125,3-133,8)	111,1 (71,4-150,8)	267,9 (228,6-307,2)	220,3 (0–525,7)	133,9 (107,2-160,6)	99,9 (0–325,8)	259,5 (255,0-264,1)	244,3 (208,4-280,3)	119,1 (116,2-122,0)	165,6 (133,8-197,5)
	**2008**	241,6 (235,4-247,7)	219,8 (169,8-269,7)	124,3 (120,2-128,5)	112,0 (74,4-149,6)	255,2 (217,1-293,4)	214,6 (0–507,9)	128,0 (102,0-153,9)	98,2 (0–311,7)	259,5 (254,9-264,0)	307,8 (267,0-348,5)	117,6 (114,7-120,5)	132,8 (105,2-160,4)
**Cirrhosis and chronic liver diseases**	**2006**	104,7 (100,4-109,0)	59,0 (39,5-78,4)	49,0 (46,2-51,9)	40,7 (22,9-58,4)	106,4 (80,6-132,1)	89,1 (0–220,1)	50,8 (33,0-68,6)	53,1 (0–159,6)	222,9 (218,6-227,2)	159,2 (140,7-177,6)	138,8 (135,5-142,1)	135,2 (115,1-155,4)
	**2007**	86,5 (82,6-90,4)	49,5 (33,9-65,1)	39,4 (36,8-42,0)	34,2 (18,7-49,7)	93,0 (69,0-117,1)	97,3 (0–247,7)	44,6 (27,9-61,3)	48,1 (0–149,2)	202,7 (198,6-206,9)	136,6 (119,5-153,6)	121,6 (118,6-124,7)	100,4 (84,0-116,8)
	**2008**	80,1 (76,4-83,9)	58,1 (39,2-77,0)	33,5 (31,1-35,9)	34,0 (20,9-47,1)	83,1 (60,4-105,8)	92,3 (0–233,8)	38,3 (22,8-53,8)	46,0 (0–152,9)	172,5 (168,7-176,3)	126,1 (107,9-144,3)	101,6 (98,8-104,4)	110,2 (91,9-128,5)

**Table 4:** **Cancers - standardized hospitalization rates for European population (per 100.000), with 95% CI, stratified for macro-area and year**

		**Northern Italy**		**Central Italy**		**Southern Italy**	
		**Italians**	**Immigrants**	**Italians**	**Immigrants**	**Italians**	**Immigrants**
**Cervical cancer**	**2006**	18,6 (16,0-21,2)	26,5 (10,0-43,1)	15,3 (1,6-28,9)	32,9 (0–168,0)	10,6 (9,2-11,9)	20,7 (10,7-30,8)
	**2007**	17,8 (15,2-20,3)	24,0 (9,6-38,4)	14,4 (1,1-27,7)	31,2 (0–146,1)	11,1 (9,8-12,4)	25,5 (14,4-36,6)
	**2008**	17,0 (14,5-19,5)	23,5 (11,7-35,4)	15,8 (1,9-29,7)	28,1 (0–109,8)	11,6 (10,2-13,0)	25,6 (15,7-35,4)
**Mastectomy**	**2006**	242,5 (233,9-251,1)	102,1 (38,2-166,0)	202,4 (154,0-250,7)	128,2 (0–562,7)	135,5 (130,9-140,2)	105,6 (62,5-148,6)
	**2007**	240,4 (231,9-248,9)	143,6 (69,8-217,4)	198,4 (150,7-246,1)	134,1 (0–577,6)	138,4 (133,7-143,0)	159,9 (107,5-212,3)
	**2008**	97,0 (91,4-102,6)	40,8 (15,5-66,2)	81,4 (50,5-112,3)	47,9 (0–220,6)	54,5 (51,6-57,4)	47,3 (27,7-67,0)

**Table 5:** **Appendectomy - standardized hospitalization rates for European population (per 100.000), with 95% CI, stratified for macro-area and year**

	**Northern Italy**				**Central Italy**				**Southern Italy**			
	**Males**		**Females**		**Males**		**Females**		**Males**		**Females**	
	**Italians**	**Immigrants**	**Italians**	**Immigrants**	**Italians**	**Immigrants**	**Italians**	**Immigrants**	**Italians**	**Immigrants**	**Italians**	**Immigrants**
**2006**	106,9 (101,9-111,9)	138,2 (117,3-159,1)	99,6 (94,8-104,4)	125,3 (104,9-145,7)	91,7 (65,2-118,1)	121,8 (0–247,5)	79,3 (54,6-104,1)	103,5 (0–215,0)	84,8 (81,9-87,8)	119,0 (108,5-129,6)	90,2 (87,1-93,2)	127,2 (114,8-139,6)
**2007**	100,2 (95,3-105,0)	128,7 (110,0-147,3)	92,7 (88,1-97,3)	111,7 (94,2-129,1)	82,3 (57,3-107,2)	110,2 (5,0-215,4)	71,9 (48,4-95,3)	93,6 (0–197,5)	80,4 (77,5-83,3)	113,1 (100,5-125,7)	81,3 (78,4-84,2)	106,4 (92,2-120,7)
**2008**	96,7 (92,0-101,5)	131,4 (112,2-150,6)	86,0 (81,5-90,5)	107,0 (90,2-123,7)	78,8 (54,4-103,2)	111,0 (0–225,2)	68,1 (45,2-91,0)	86,8 (0–178,3)	76,4 (73,6-79,2)	107,7 (95,0-120,4)	74,4 (71,6-77,1)	79,7 (71,7-87,6)

A decrease in hospitalization rates for appendectomy was found in all macro-areas, in both genders and citizenships. Notwithstanding, the decrease was significant only for Italians in Northern and Southern Regions.

Details on differences linked to migrant status, gender and geographical macro-area are as follows.

### Differences related to migrant status

Immigrants showed significantly higher hospitalization rates for stroke as compared to Italians in Southern Italy. Regarding myocardial infarction, immigrants had generally lower rates if compared to Italians even though differences were not significant. With regard to chronic liver diseases, hospitalization rates of immigrants were significantly lower than Italians in Northern and Southern Italy.

Hospitalization rates for mastectomy were generally lower for immigrants, even though differences were significant only in Northern Italy. In contrast, cervical cancer hospitalization rates were generally higher among immigrants, in some cases more than two-fold. Nonetheless, differences were statistically significant in Southern Italy only.

Finally, immigrants had significantly higher hospitalization rates than Italians for appendectomy.

### Differences related to gender

With regards to gender, stroke hospitalization rates were similar in males and females. As for myocardial infarction, males had significantly higher hospitalization rates than females. This was verified also for chronic liver diseases, but in Italians only. For appendectomy, males had generally higher rates than females but differences did not reach the significance but for Southern Italy.

### Differences related to geographical macro-areas

Hospitalization rates for stroke, myocardial infarction and chronic liver diseases were significantly lower in Northern Italy than Southern Italy.

On the contrary, hospitalization rates for mastectomy were higher in Northern Italy for Italians only whilst hospitalization rates for cervical cancer did not show significant trends.

With respect to appendectomy, significantly higher rates were observed in Northern Italy for Italians (Table 5).

Differences between Central and Northern/Southern Italy were more difficult to assess because of the high variability of data in Central Italy.

## Discussion

### Main findings

This work was primarily aimed at investigating differences in the quality of health care likely to be associated to migrant status, gender and geographical macro-area. At the same time, the study investigated time trends.

With respect to the latter, a general decrease in total as well as disease-specific hospitalization rates was observed. This result is quite important and could suggest an improvement in quality of care. In particular, a better control of inappropriateness, which is a dimension of quality [16], was highlighted by results. Furthermore, the significant reduction in hospitalization for chronic liver diseases and mastectomy may underline an improvement of primary and secondary prevention interventions over time. Notwithstanding, the decrease of hospitalization rates over time may also be the result of changes in the Italian NHS due to economic constraints which led to a reduction of total number of hospital beds (from 3,3 to 3,0 per 1.000 inhabitants from 2004 to 2008 [24]).

With respect to migrant status, important differences resulted from the analysis. First of all, it should be observed that total hospitalization rates were higher for Italians than immigrants. This may suggest constraints, in particular, in the access to health services as well as differences in the health status between Italians and immigrants. In fact, literature shows that immigrants are often healthier than native-born [25].

Nevertheless, looking at specific diseases, peculiarities may be appreciated. In fact, summarizing results yielded by the study, immigrants showed higher rates of hospitalizations with regard to stroke, cervical cancer and appendectomy with some differences with respect to geographical area. These results may hide a deficiency in primary and secondary prevention (e.g. lack/inefficacy of educational campaigns in immigrants; low access to cervical cancer screening by immigrant women). In particular, as far as stroke is concerned, the limited access to preventive medicine interventions, reported also in other studies on “migrant health status” [26], may result in an unequal distribution of risk factors for cardiovascular diseases, such as smoking, stress, unhealthy diet and hypertension. A higher incidence of cardiovascular diseases was also observed in immigrant population in comparison to native-born in Denmark. Interestingly, the differences between immigrant and Danish population were reduced after the adjustment for socio-economic factors such as income, employment, housing and education [27]. With respect to cervical cancer, the higher hospitalization rates of immigrant women may be probably due to a greater exposure to Human Papilloma Virus infection [28,29] and to a limited access to screening [30-33]. Finally, regarding appendectomy, the higher hospitalizations rates of immigrants may underline a greater risk for inappropriateness due to a lower level of quality of care. In fact, the datum could be more likely attributed to unnecessary interventions instead of a higher occurrence of appendicitis among immigrants since the disease is reported to be lower in immigrants mainly because of factors which depend on dietary habits [22].

On the contrary, hospitalization rates were lower for immigrants than Italians with respect to chronic liver diseases and mastectomy with some differences due to geographical area. This may suggest a more attention paid in the primary care of liver chronic conditions as well as cancer in Italians than immigrants. In particular, with respect to mastectomy, higher hospitalization rates of Italians may be due to the early detection of cancer which is linked to the access to screening programs [32]. Alongside, it should be also observed that some risk factors for breast cancer, such as nulliparity, oral contraceptives use and artificial feeding, may be unequally distributed among immigrants and Italians so justifying in part results. The higher rates of hospitalizations for chronic liver diseases of Italians may be the indication of an easier access, which is also an aspect of healthcare quality, to specialized care by Italians than immigrants. With this respect, a limited access of immigrants to diagnostic and treatment services for hepatitis has been evidenced by Fasano M. *et al.* [34]. Furthermore the above-mentioned “healthy migrant effect” [35] may also play a role with respect to chronic liver diseases.

As far as gender differences are concerned, disease-specific hospitalization rates were generally higher in males than females. This result could suggest that women may face more barriers in the access to health services.

Finally, with respect to geographic trend, a North–South gradient was shown, with the highest rates of hospitalization in the Southern Italy for both immigrants and Italians, indicating a possible unequal distribution of socio-economic factors as well as inequalities in the quality of health care.

In particular, as far as chronic diseases are concerned, Southern Italy showed the highest hospitalization rates. On the other hand, Northern Italy was characterized by higher rates of hospitalizations for mastectomy and appendectomy in Italians. These differences should be carefully considered and better investigated with respect to their causes which may be related to the health care organization outside the hospitals as well as to local health and other policies.

The results which were released by this study could be different if we considered immigrants’ country of origin. Notwithstanding, in the comparison between macro-areas, results may be considered not biased because 55-60% of immigrants are from Europe and 15-20% from Africa in either Northern, Central and Southern Italy. In particular, around 40% of immigrants in all areas are from Romania, Albania and Morocco [36].

This study has some limitations. First of all, it relies only on few indicators which were considered as a proxy of the quality of health care services. As a consequence, the analysis cannot be considered thorough with respect to the different levels of health care. Furthermore, it did not take into consideration outcome indicators which are more valuable in the evaluation of the performance of health care systems. Another limitation is represented by the use of citizenship as a criterion to establish migrant status even though it should be considered more reliable than the birth place which is the alternative way used in the literature to define the migrant status. Notwithstanding, misclassification of some cases cannot be excluded also because of the use of administrative data flows. With respect to this last aspect, it should be added that administrative data flows may present missing data as well as mistakes which may not be controlled.

On the other hand, some strengths may be seen. The analysis was carried out at national level, being quite useful for national health care planning. Furthermore, it attempted to describe the quality of health care provided to immigrant population. With this respect, in contrast to other analysis which were performed on official statistics, our study relied on a new estimate of immigrant population which took into consideration also people living irregularly in our country. Indeed, generalizability of results may be considered quite good within the national context, albeit it cannot be stated the same outside Italy because of different health and immigration policies. Furthermore, the comparison of hospitalization rates between men and women as well as Italians and immigrants may be considered unbiased because of the standardization process which allowed controlling for age.

## Conclusion

In conclusion, on the basis of the evidence also, which suggests that some barriers, such as language difficulties, a poor awareness of health status and the lack of economic resources, may influence the quality of provided healthcare services [37], implications for both research and policy may be recognized. In particular, research should be promoted in the field of the assessment of reasons behind inequalities in order to support the development of tailored interventions and policies for the reduction of differences in the quality of health care.

### Ethics

In order to carry out the study anonymous aggregated data were collected from routinely administrative databases. This ensured patients’ privacy and allowed not requiring patients’ personal informed consent. For this reason, the approval of the ethics committee was not requested.

## Additional file

Additional file 1:
**Number of hospitalizations stratified for macro-area and year.**
