# Supplementary material for: Health inequalities: an analysis of hospitalizations with respect to migrant status, gender and geographical area
Source: BMC Int Health Hum Rights. 2015 Feb 7;15:2. doi: 10.1186/s12914-014-0032-9 (PMC4336515; doi:10.1186/s12914-014-0032-9)
Supplement: Additional file 1: — Number of hospitalizations stratified for macro-area and year. [file 12914_2014_32_MOESM1_ESM.docx]

|  |  | **Northern Italy** | | | | **Central Italy** | | | | **Southern Italy** | | | |
| --- | --- | --- | --- | --- | --- | --- | --- | --- | --- | --- | --- | --- | --- |
|  |  | **Males** | | **Females** | | **Males** | | **Females** | | **Males** | | **Females** | |
|  |  | **Italians** | **Immigrants** | **Italians** | **Immigrants** | **Italians** | **Immigrants** | **Italians** | **Immigrants** | **Italians** | **Immigrants** | **Italians** | **Immigrants** |
| **2006** | **0-14** | 183.729 | 25.754 | 141.801 | 19.463 | 77.129 | 9.604 | 56.901 | 6.843 | 451.241 | 13.053 | 380.022 | 13.492 |
|  | **15-24** | 80.212 | 8.105 | 84.067 | 29.709 | 35.503 | 3.563 | 38.435 | 14.292 | 423.855 | 14.236 | 658.896 | 35.905 |
|  | **25-49** | 363.608 | 36.270 | 649.528 | 97.853 | 160.891 | 15.783 | 280.126 | 44.754 | 811.461 | 25.333 | 1.215.218 | 61.426 |
|  | **50-64** | 422.470 | 8.132 | 384.261 | 9.482 | 172.469 | 3.453 | 159.031 | 4.545 | 945.752 | 6.728 | 898.265 | 9.602 |
|  | **65+** | 832.296 | 5.141 | 868.143 | 5.675 | 394.143 | 2.055 | 395.300 | 2.270 | 1.008.243 | 3.840 | 991.856 | 5.127 |
| **2007** | **0-14** | 173.030 | 26.496 | 131.926 | 20.639 | 75.531 | 9.742 | 55.425 | 7.335 | 346.460 | 11.565 | 263.596 | 8.755 |
|  | **15-24** | 76.422 | 8.686 | 79.198 | 30.413 | 35.466 | 3.911 | 37.122 | 14.697 | 153.902 | 6.035 | 187.324 | 16.660 |
|  | **25-49** | 342.496 | 37.745 | 617.932 | 103.058 | 156.974 | 16.307 | 275.581 | 48.280 | 515.656 | 23.760 | 929.057 | 59.485 |
|  | **50-64** | 404.694 | 9.270 | 359.035 | 10.500 | 172.466 | 4.027 | 157.790 | 5.252 | 546.562 | 6.052 | 508.363 | 9.069 |
|  | **65+** | 801.257 | 5.436 | 807.144 | 6.012 | 395.302 | 2.213 | 396.351 | 2.520 | 972.705 | 3.987 | 937.942 | 5.274 |
| **2008** | **0-14** | 168.709 | 28.227 | 128.143 | 21.040 | 73.557 | 10.718 | 54.604 | 8.096 | 330.501 | 12.188 | 252.071 | 9.159 |
|  | **15-24** | 75.035 | 9.096 | 76.300 | 31.503 | 35.021 | 4.260 | 35.871 | 15.723 | 148.240 | 5.828 | 178.235 | 16.355 |
|  | **25-49** | 330.146 | 39.568 | 603.420 | 108.813 | 153.369 | 17.347 | 269.137 | 52.197 | 487.232 | 22.957 | 896.285 | 59.148 |
|  | **50-64** | 391.723 | 10.158 | 346.749 | 12.238 | 169.555 | 4.641 | 156.916 | 6.376 | 533.231 | 6.343 | 491.467 | 9.373 |
|  | **65+** | 799.684 | 5.748 | 803.420 | 6.665 | 395.728 | 2.597 | 398.883 | 3.055 | 952.471 | 4.305 | 916.936 | 5.646 |

Number of total hospitalizations

Number of hospitalizations for stroke

|  |  | **Northern Italy** | | | | **Central Italy** | | | | **Southern Italy** | | | |
| --- | --- | --- | --- | --- | --- | --- | --- | --- | --- | --- | --- | --- | --- |
|  |  | **Males** | | **Females** | | **Males** | | **Males** | | **Females** | | **Males** | |
|  |  | **Italians** | **Immigrants** | **Italians** | **Italians** | **Italians** | **Immigrants** | **Italians** | **Immigrants** | **Italians** | **Immigrants** | **Italians** | **Immigrants** |
| **2006** | **0-14** | 50 | 4 | 24 | 3 | 15 | 4 | 12 | 4 | 66 | 4 | 57 | 0 |
|  | **15-24** | 59 | 6 | 48 | 5 | 24 | 5 | 20 | 3 | 131 | 15 | 100 | 6 |
|  | **25-49** | 1.244 | 144 | 1.019 | 139 | 532 | 77 | 413 | 60 | 2.008 | 162 | 1.510 | 110 |
|  | **50-64** | 4.054 | 164 | 2.003 | 95 | 1.861 | 65 | 1.022 | 72 | 6.343 | 102 | 3.322 | 110 |
|  | **65+** | 16.519 | 164 | 21.129 | 198 | 9.860 | 60 | 11.490 | 55 | 23.980 | 123 | 28.287 | 146 |
| **2007** | **0-14** | 54 | 10 | 27 | 3 | 18 | 2 | 6 | 5 | 39 | 2 | 39 | 4 |
|  | **15-24** | 62 | 7 | 60 | 7 | 22 | 1 | 24 | 5 | 86 | 3 | 55 | 7 |
|  | **25-49** | 1.280 | 181 | 934 | 124 | 562 | 78 | 437 | 64 | 1.287 | 112 | 1.033 | 87 |
|  | **50-64** | 3.956 | 176 | 1.981 | 108 | 1.749 | 65 | 901 | 74 | 4.416 | 88 | 2.231 | 84 |
|  | **65+** | 16.464 | 169 | 21.121 | 202 | 9.353 | 67 | 11.153 | 60 | 18.012 | 114 | 20.685 | 119 |
| **2008** | **0-14** | 57 | 15 | 55 | 6 | 16 | 4 | 13 | 8 | 42 | 3 | 48 | 1 |
|  | **15-24** | 121 | 14 | 68 | 10 | 24 | 6 | 24 | 3 | 79 | 8 | 55 | 5 |
|  | **25-49** | 1.452 | 199 | 1.381 | 134 | 525 | 94 | 433 | 79 | 1.214 | 98 | 1.007 | 65 |
|  | **50-64** | 3.862 | 181 | 1.911 | 145 | 1.721 | 80 | 923 | 72 | 4.432 | 94 | 2.334 | 60 |
|  | **65+** | 15.913 | 178 | 20.405 | 195 | 9.289 | 81 | 11.103 | 75 | 17.302 | 105 | 20.044 | 126 |

Number of hospitalizations for myocardial infarction

|  |  | **Northern Italy** | | | | **Central Italy** | | | | **Southern Italy** | | | |
| --- | --- | --- | --- | --- | --- | --- | --- | --- | --- | --- | --- | --- | --- |
|  |  | **Males** | | **Females** | | **Males** | | **Males** | | **Females** | | **Males** | |
|  |  | **Italians** | **Immigrants** | **Italians** | **Immigrants** | **Italians** | **Immigrants** | **Italians** | **Immigrants** | **Italians** | **Immigrants** | **Italians** | **Immigrants** |
| **2006** | **0-14** | 7 | 0 | 0 | 0 | 0 | 0 | 0 | 0 | 15 | 0 | 4 | 0 |
|  | **15-24** | 11 | 2 | 3 | 0 | 12 | 2 | 1 | 1 | 1.036 | 27 | 142 | 3 |
|  | **25-49** | 3.384 | 355 | 564 | 60 | 1.570 | 141 | 233 | 27 | 6.112 | 102 | 1.148 | 19 |
|  | **50-64** | 10.795 | 302 | 2.456 | 109 | 5.113 | 143 | 1.090 | 57 | 13.804 | 87 | 5.369 | 32 |
|  | **65+** | 21.184 | 240 | 17.721 | 133 | 9.998 | 100 | 7.517 | 82 | 13.512 | 58 | 9.579 | 42 |
| **2007** | **0-14** | 0 | 0 | 0 | 0 | 1 | 0 | 0 | 0 | 3 | 0 | 2 | 0 |
|  | **15-24** | 19 | 3 | 4 | 1 | 10 | 0 | 1 | 0 | 21 | 0 | 2 | 0 |
|  | **25-49** | 3.241 | 373 | 626 | 64 | 1.480 | 148 | 281 | 28 | 3.062 | 94 | 536 | 14 |
|  | **50-64** | 10.561 | 372 | 2.430 | 113 | 4.848 | 150 | 1.122 | 72 | 9.229 | 93 | 2.016 | 52 |
|  | **65+** | 21.038 | 230 | 17.850 | 140 | 10.293 | 104 | 7.847 | 64 | 13.365 | 65 | 9.905 | 53 |
| **2008** | **0-14** | 0 | 0 | 0 | 0 | 0 | 0 | 1 | 0 | 7 | 0 | 2 | 0 |
|  | **15-24** | 4 | 5 | 2 | 0 | 16 | 0 | 0 | 0 | 21 | 1 | 2 | 0 |
|  | **25-49** | 3.139 | 394 | 568 | 57 | 1.433 | 173 | 220 | 31 | 3.054 | 105 | 525 | 32 |
|  | **50-64** | 10.347 | 402 | 2.321 | 139 | 4.798 | 165 | 1.082 | 52 | 9.325 | 96 | 2.124 | 36 |
|  | **65+** | 21.289 | 209 | 17.359 | 154 | 9.702 | 108 | 7.643 | 77 | 13.579 | 92 | 9.769 | 45 |

Number of hospitalizations for cirrhosis and chronic hepatitis

|  |  | **Northern Italy** | | | | **Central Italy** | | | | **Southern Italy** | | | |
| --- | --- | --- | --- | --- | --- | --- | --- | --- | --- | --- | --- | --- | --- |
|  |  | **Males** | | **Females** | | **Males** | | **Males** | | **Females** | | **Males** | |
|  |  | **Italians** | **Immigrants** | **Italians** | **Immigrants** | **Italians** | **Immigrants** | **Italians** | **Immigrants** | **Italians** | **Immigrants** | **Italians** | **Immigrants** |
| 2006 | 0-14 | 94 | 31 | 110 | 19 | 167 | 51 | 198 | 29 | 277 | 17 | 227 | 11 |
|  | 15-24 | 168 | 53 | 118 | 36 | 192 | 41 | 104 | 18 | 3.459 | 95 | 1.312 | 89 |
|  | 25-49 | 4.847 | 465 | 1.810 | 258 | 2.187 | 253 | 786 | 161 | 11.210 | 276 | 5.174 | 229 |
|  | 50-64 | 4.974 | 93 | 2.605 | 64 | 1.882 | 56 | 968 | 56 | 9.207 | 54 | 7.562 | 55 |
|  | 65+ | 3.719 | 14 | 2.150 | 17 | 1.347 | 7 | 873 | 7 | 4.741 | 9 | 4.496 | 14 |
| 2007 | 0-14 | 67 | 26 | 70 | 22 | 173 | 38 | 187 | 36 | 109 | 4 | 92 | 3 |
|  | 15-24 | 116 | 41 | 66 | 28 | 140 | 56 | 107 | 19 | 421 | 43 | 211 | 18 |
|  | 25-49 | 3.718 | 454 | 1.430 | 220 | 1.860 | 243 | 695 | 151 | 7.882 | 259 | 2.929 | 202 |
|  | 50-64 | 4.332 | 106 | 2.125 | 70 | 1.752 | 67 | 790 | 48 | 6.764 | 41 | 5.150 | 55 |
|  | 65+ | 3.248 | 9 | 1.799 | 17 | 1.217 | 17 | 829 | 9 | 4.668 | 11 | 4.064 | 11 |
| 2008 | 0-14 | 80 | 32 | 94 | 31 | 166 | 22 | 165 | 22 | 76 | 2 | 79 | 5 |
|  | 15-24 | 83 | 46 | 60 | 27 | 118 | 40 | 94 | 23 | 321 | 33 | 212 | 23 |
|  | 25-49 | 3.275 | 458 | 1.168 | 271 | 1.644 | 264 | 600 | 125 | 6.558 | 207 | 2.405 | 204 |
|  | 50-64 | 4.157 | 110 | 1.726 | 85 | 1.525 | 87 | 671 | 41 | 5.969 | 42 | 4.202 | 53 |
|  | 65+ | 3.074 | 23 | 1.640 | 13 | 1.162 | 15 | 711 | 16 | 4.068 | 15 | 3.601 | 17 |

Numbers of hospitalizations for cervical cancer

|  |  | **Northern Italy** | | **Central Italy** | | **Suthern Italy** | |
| --- | --- | --- | --- | --- | --- | --- | --- |
|  |  | **Immingrants** | **Italians** | **Immingrants** | **Immingrants** | **Italians** | **Immingrants** |
| **2006** | **0-14** | 0 | 0 | 0 | 0 | 2 | 0 |
|  | **15-24** | 104 | 20 | 16 | 7 | 117 | 11 |
|  | **25-49** | 2.687 | 475 | 848 | 175 | 1.637 | 202 |
|  | **50-64** | 901 | 79 | 321 | 28 | 874 | 64 |
|  | **65+** | 680 | 11 | 261 | 6 | 628 | 12 |
| **2007** | **0-14** | 0 | 0 | 0 | 0 | 1 | 0 |
|  | **15-24** | 97 | 16 | 19 | 6 | 48 | 6 |
|  | **25-49** | 2.873 | 569 | 767 | 216 | 1.672 | 306 |
|  | **50-64** | 953 | 99 | 327 | 42 | 846 | 68 |
|  | **65+** | 807 | 11 | 276 | 5 | 617 | 14 |
| **2008** | **0-14** | 0 | 0 | 0 | 0 | 0 | 0 |
|  | **15-24** | 115 | 22 | 20 | 7 | 94 | 7 |
|  | **25-49** | 2.716 | 633 | 871 | 245 | 1.820 | 305 |
|  | **50-64** | 867 | 93 | 277 | 53 | 901 | 89 |
|  | **65+** | 3.698 | 748 | 1.168 | 305 | 2.815 | 401 |

Numbers of hospitalizations for mastectomy

|  |  | **Northern Italy** | | **Central Italy** | | **Suthern Italy** | |
| --- | --- | --- | --- | --- | --- | --- | --- |
|  |  | **Italians** | **Immingrants** | **Italians** | **Immingrants** | **Italians** | **Immingrants** |
| **2006** | **0-14** | 0 | 0 | 0 | 0 | 2 | 0 |
|  | **15-24** | 3 | 1 | 0 | 0 | 20 | 0 |
|  | **25-49** | 33 | 2 | 23 | 3 | 285 | 10 |
|  | **50-64** | 5.418 | 232 | 2.192 | 133 | 4.487 | 189 |
|  | **65+** | 7.598 | 133 | 3.372 | 83 | 5.311 | 79 |
| **2007** | **0-14** | 0 | 0 | 0 | 0 | 0 | 0 |
|  | **15-24** | 5 | 1 | 2 | 2 | 7 | 1 |
|  | **25-49** | 58 | 8 | 31 | 1 | 67 | 2 |
|  | **50-64** | 6.378 | 320 | 2.120 | 143 | 4.297 | 165 |
|  | **65+** | 9.255 | 228 | 3.189 | 87 | 5.146 | 86 |
| **2008** | **0-14** | 0 | 0 | 0 | 0 | 0 | 0 |
|  | **15-24** | 0 | 0 | 1 | 0 | 2 | 0 |
|  | **25-49** | 28 | 4 | 14 | 2 | 54 | 4 |
|  | **50-64** | 5.446 | 272 | 2.122 | 140 | 4.446 | 226 |
|  | **65+** | 7.868 | 178 | 3.311 | 125 | 5.421 | 113 |

Numbers of hospitalizations for appendectomy

|  |  | **Northern Italy** | | | | **Central Italy** | | | | **Southern Italy** | | | |
| --- | --- | --- | --- | --- | --- | --- | --- | --- | --- | --- | --- | --- | --- |
|  |  | **Males** | | **Females** | | **Males** | | **Males** | | **Females** | | **Males** | |
|  |  | **Italians** | **Immigrants** | **Italians** | **Immigrants** | **Italians** | **Immigrants** | **Italians** | **Immigrants** | **Italians** | **Immigrants** | **Italians** | **Immigrants** |
| **2006** | **0-14** | 3.906 | 404 | 3.150 | 295 | 1.466 | 109 | 1.101 | 90 | 4.208 | 85 | 4.300 | 92 |
|  | **15-24** | 2.647 | 551 | 3.513 | 587 | 1.000 | 180 | 1.266 | 214 | 3.021 | 130 | 4.561 | 180 |
|  | **25-49** | 3.370 | 1.088 | 3.015 | 903 | 1.224 | 351 | 1.049 | 277 | 2.246 | 140 | 1.977 | 170 |
|  | **50-64** | 819 | 53 | 534 | 51 | 310 | 27 | 190 | 22 | 572 | 12 | 361 | 14 |
|  | **65+** | 608 | 10 | 477 | 14 | 219 | 4 | 170 | 5 | 295 | 1 | 211 | 3 |
| **2007** | **0-14** | 3.548 | 409 | 2.894 | 339 | 1.312 | 127 | 992 | 72 | 3.222 | 65 | 2.962 | 48 |
|  | **15-24** | 2.461 | 570 | 3.259 | 580 | 873 | 187 | 1.143 | 222 | 2.140 | 98 | 3.433 | 108 |
|  | **25-49** | 3.241 | 1.134 | 2.775 | 879 | 1.116 | 353 | 984 | 277 | 1.932 | 142 | 1.777 | 150 |
|  | **50-64** | 803 | 70 | 528 | 66 | 317 | 25 | 175 | 29 | 468 | 14 | 265 | 16 |
|  | **65+** | 594 | 10 | 462 | 13 | 220 | 2 | 146 | 6 | 296 | 5 | 221 | 8 |
| **2008** | **0-14** | 3.376 | 425 | 2.710 | 321 | 1.106 | 135 | 922 | 90 | 2.916 | 63 | 2.544 | 41 |
|  | **15-24** | 2.423 | 558 | 3.024 | 545 | 924 | 214 | 1.082 | 212 | 2.053 | 90 | 3.197 | 122 |
|  | **25-49** | 3.044 | 1.156 | 2.503 | 877 | 1.123 | 325 | 908 | 275 | 1.874 | 144 | 1.590 | 146 |
|  | **50-64** | 822 | 78 | 470 | 75 | 319 | 29 | 192 | 31 | 448 | 13 | 275 | 6 |
|  | **65+** | 586 | 15 | 454 | 15 | 199 | 6 | 148 | 5 | 303 | 6 | 229 | 1 |
